# Protocol for multicentre comparison of interictal high-frequency oscillations as a predictor of seizure freedom

**DOI:** 10.1093/braincomms/fcac151

**Published:** 2022-06-09

**Authors:** Vasileios Dimakopoulos, Jean Gotman, William Stacey, Nicolás von Ellenrieder, Julia Jacobs, Christos Papadelis, Jan Cimbalnik, Gregory Worrell, Michael R Sperling, Maike Zijlmans, Lucas Imbach, Birgit Frauscher, Johannes Sarnthein

**Affiliations:** Klinik für Neurochirurgie, UniversitätsSpital Zürich, Universität Zürich, Zürich, Switzerland; Montreal Neurological Institute & Hospital, McGill University, Montreal, Quebec, Canada; Department of Neurology and Department of Biomedical Engineering, University of Michigan, Ann Arbor, Michigan, MI, USA; Montreal Neurological Institute & Hospital, McGill University, Montreal, Quebec, Canada; Alberta Children’s Hospital, University of Calgary, Calgary, Canada; Cook Children’s Health Care System, Fort Worth, TX, USA; St. Anne’s University Hospital, Brno, Czech Republic; Department of Neurology, Mayo Clinic, Rochester, MN, USA; Department of Neurology, Jefferson University Hospitals, Philadelphia, PA, USA; University Medical Center, Utrecht, and Stichting Epilepsie Instellingen Nederland (SEIN), Utrecht, The Netherlands; Schweizerisches Epilepsie Zentrum, Zurich, Switzerland; Montreal Neurological Institute & Hospital, McGill University, Montreal, Quebec, Canada; Klinik für Neurochirurgie, UniversitätsSpital Zürich, Universität Zürich, Zürich, Switzerland

**Keywords:** ripples, fast ripples, automated detection, epilepsy surgery, intracranial EEG

## Abstract

In drug-resistant focal epilepsy, interictal high-frequency oscillations (HFOs) recorded from intracranial EEG (iEEG) may provide clinical information for delineating epileptogenic brain tissue. The iEEG electrode contacts that contain HFO are hypothesized to delineate the epileptogenic zone; their resection should then lead to postsurgical seizure freedom. We test whether our prospective definition of clinically relevant HFO is in agreement with postsurgical seizure outcome. The algorithm is fully automated and is equally applied to all data sets. The aim is to assess the reliability of the proposed detector and analysis approach.

We use an automated data-independent prospective definition of clinically relevant HFO that has been validated in data from two independent epilepsy centres. In this study, we combine retrospectively collected data sets from nine independent epilepsy centres. The analysis is blinded to clinical outcome. We use iEEG recordings during NREM sleep with a minimum of 12 epochs of 5 min of NREM sleep. We automatically detect HFO in the ripple (80–250 Hz) and in the fast ripple (250–500 Hz) band. There is no manual rejection of events in this fully automated algorithm. The type of HFO that we consider clinically relevant is defined as the simultaneous occurrence of a fast ripple and a ripple. We calculate the temporal consistency of each patient’s HFO rates over several data epochs within and between nights. Patients with temporal consistency <50% are excluded from further analysis. We determine whether all electrode contacts with high HFO rate are included in the resection volume and whether seizure freedom (ILAE 1) was achieved at ≥2 years follow-up. Applying a previously validated algorithm to a large cohort from several independent epilepsy centres may advance the clinical relevance and the generalizability of HFO analysis as essential next step for use of HFO in clinical practice.

## Teaser

High-frequency oscillations (HFOs) have long been discussed as biomarkers for epileptogenic tissue. Dimakopoulos et al. propose a prospective multicentre study to validate whether HFOs in intracranial EEG recorded during sleep may serve to predict the seizure outcome after epilepsy surgery.

## Graphical Abstract

**Graphical abstract:**
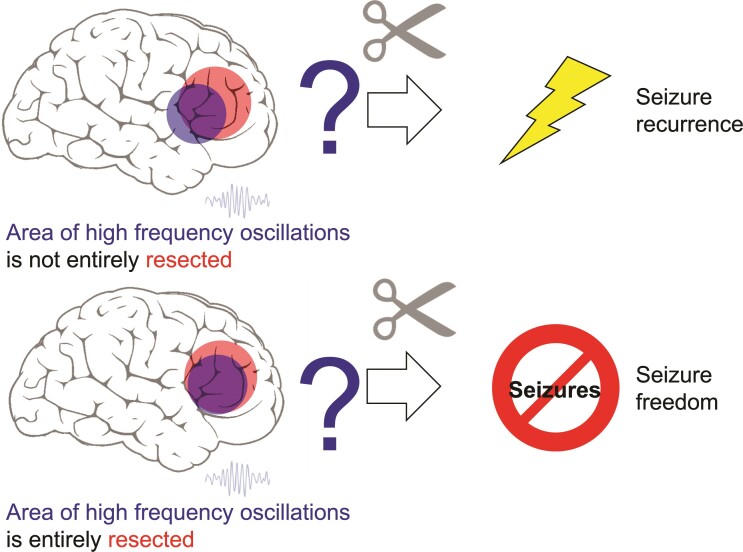

## Introduction

### Background and rationale

Drug-resistant focal epilepsy is a common condition. In selected patients, surgical resection of the epileptogenic zone is the treatment of choice and may eliminate the occurrence of seizures completely.^1^ The epileptogenic zone may be defined as the minimum brain area whose resection leads to freedom from seizures.^2^ The aim of epilepsy surgery is seizure freedom (ILAE 1).^3^ Preoperative diagnostic workup may involve recording of intracranial EEG (iEEG) to determine the seizure onset zone (SOZ) as an estimate for the epileptogenic zone.^4,5^ Since seizures are usually rare events during the limited duration of the iEEG recording, it would be advantageous to determine the epileptogenic zone during the interictal period. In this approach, the traditional analysis of interictal epileptic discharges has a high sensitivity but low specificity as a marker of epileptogenic tissue.^4,6^

Another marker, high-frequency oscillation (HFO), may have the potential to be a clinical asset for delineating epileptogenic brain areas and identifying successful surgical treatments. These oscillatory events can be found in the frequency range between 80–500 Hz. HFO are sub-classified in ripples (80–250 Hz) and fast ripples (FR, 250–500 Hz). Interictal HFO are discussed to be more specific than interictal spikes in localizing the SOZ or being in agreement with seizure outcome in several studies.^7–13^ Many studies present HFO rates in relation to SOZ electrodes (for a review see^14^). Fewer studies analyse the resection of interictal HFO, marked prospectively, to test agreement with postsurgical seizure freedom.^10,11,15–20^

Investigations in the clinical relevance of HFO have been facilitated by automated or semi-automated detection algorithms.^18^ Here we apply a fully automated definition of HFO, which we built on visual markings in a data set of the Montreal Neurological Institute.^21^ This definition of HFO was then embedded in an algorithm that used the temporal consistency of HFO occurrence in each patient to gauge the validity of the outcome prediction in that patient, and the algorithm was applied to a Zurich cohort.^17^ Next, the algorithm was applied on independently recorded data from Geneva where a blinded analysis was in agreement with seizure outcome.^22^ The algorithm thus provides a prospective definition of a clinically relevant HFO.

Over the last years, many ways have evolved on how to define an HFO. From the perspective of reliability, automated definitions are preferred over semi-automated definitions or expert visual markings. Among automated definitions, some show lower temporal consistency^23^ than others,^17,22^ which has triggered a discussion on how to define a clinically relevant HFO.^24,25^ When testing for the clinical relevance of HFO, these may be masked by potential confounders, e.g. physiological HFO related to sensory-motor function^26,27^ or cognitive activity^28,29^ or effects of high-pass filtering.^30^ The type of HFO that we consider clinically relevant is defined as the simultaneous occurrence of a fast ripple and ripple (FRandR), as they have been proven more specific at delineating the epileptogenic zone than FR or ripples alone.^17^ The FRandR show a strong association with interictal spikes.^13,22^

For the widespread application of HFO in clinical use, it is essential to have an approach that works independently of the data set. It was one of the priorities identified at the international HFO workshop in Freiburg^31^ to obtain a consensus on a detector and its settings. This aim requires assessing the reliability of an automated HFO detector and analysis approach. Herein lies the novelty of our proposed study protocol.

In the proposed study, we apply the same HFO algorithm to all iEEG recorded in the study centres. The study aims at validating the algorithm and protocols but not comparing HFO detectors. The analysis is blind with respect to the seizure outcome of epilepsy surgery. We compare the HFO area with the brain resection volume (RV) and test the agreement with the seizure outcome in individual patients to evaluate the clinical relevance of the algorithm for HFO analysis. Applying a previously validated algorithm to a large cohort from several independent epilepsy centres has the aim to advance the clinical relevance and the generalizability of HFO analysis as essential next step for use of HFO in clinical practice.

### Study objectives and hypotheses

The primary objective of this study is to investigate if resection of the HFO area is in agreement with seizure freedom in a large cohort of patients. Conversely, if at least one channel of the HFO area is not resected, the patient will suffer from seizure recurrence. We apply the automated detection of the HFO with a prospective definition of the events of interest (FRandR). The two previous pilot studies had small cohorts with moderate seizure freedom rates.^17,22^ The HFO analysis in a large cohort may corroborate the previous findings and establish that fully automated HFO analysis is indeed working and can be easily implemented in various clinical settings.

## Materials and methods

### Study design

In this research protocol, we investigate the predictive power of HFO rate against seizure outcome. If electrode contacts with high HFO rate (‘HFO area’) are not entirely included in the RV, the analysis expects seizure recurrence after epilepsy surgery. The patient data is provided by several independent epilepsy centres (Table 1). The HFO analysis is carried out by researchers that are blinded with respect to the seizure outcome after epilepsy surgery. HFO are defined by the automated detector (https://github.com/ZurichNCH/Automatic-High-Frequency-Oscillation-Detector).^17,22^ Given the fully automated application of the algorithm, the definition of the HFO is prospective. Given the blinded study design, the validation of HFO detection is pseudo-prospective.

**Table 1:** Study centres and patient number

i	Study centre	Patients
1	Schweizerisches Epilepsie-Zentrum^a^	15
2	Montreal Neurological Institute and Hospital	30
3	University of Michigan	30
4	Alberta Children’s Hospital	30
5	Cook Children’s Health Care System, Fort Worth	30
6	St. Anne’s University Hospital Brno	30
7	Mayo Clinic Rochester	30
8	Jefferson University Hospitals	30
9	University Medical Center Utrecht	30
	total	255

The minimum number of patients at each epilepsy centre that fulfil the inclusion criteria and will be included in the study.

^a^ Patients included in the original two studies validating this method are not included here.

### Inclusion criteria

We include patients with drug-resistant focal epilepsy of all ages who (i) underwent invasive EEG recordings with subdural and/or depth electrodes as part of their presurgical evaluation,^4^ (ii) underwent epilepsy surgery aiming at seizure freedom after resection of a single focus, and (iii) and the postsurgical seizure outcome was determined by follow-up visits at ≥ 2 years.^32^ According to the International League Against Epilepsy (ILAE) Classification^3^, we classify outcome into seizure freedom (ILAE 1) or recurrent seizures (ILAE 2–6). Outcome data that are assessed with the Engel scale are mapped to ILAE 1 or ILAE 2–6, respectively.

To participate in the study, each study centre (Table 1) provides for HFO analysis:

1. Data from ≥ 30 consecutive patients
2. Data recorded with ≥ 2000 Hz sampling rate
3. Sleep data from ≥ 2 nights, excluding the first night after implantation to avoid anaesthesia and implantation effects
4. Identification of 5 min epochs of NREM sleep
5. At least 12 epochs per patient
6. Epochs are at least 1 h after/before a focal to bilateral tonic clonic, focal impaired awareness or focal aware seizure, or 0.5 h in the case of purely electrographic seizures
7. Documentation of non-cephalic or artefact-ridden channels to be excluded from analysis

After the HFO analysis and for its validation, each study centre provides documentation of:

1. Patient’s pathology, age and gender
2. Electrode contacts that are contained in the resection volume (RV)
3. Electrode contacts that are located in sensory-motor or occipital or frontal cortex
4. Seizure outcome (ILAE 1 or ILAE 2-6) at follow-up ≥ 2 years
5. Given that the analysis and seizure outcome of 20 patients has been published recently,^17^ Schweizerisches Epilepsie-Zentrum will participate with a reduced number of remaining patients in their database fulfilling the inclusion criteria.

### Statistical analysis

We use non-parametric permutation tests for statistical hypothesis testing. We estimate the 95% confidence intervals (CI) of proportions by the binomial method. All statistical analyses are performed in Matlab. Statistical significance is established at *P* < 0.05.

### Ethical considerations

The study was registered at www.clinicaltrials.gov (NCT05332990). Each study centre seeks ethics approval by their local ethics committee. All data is pseudo-nonymized before sharing. Each patients is identified by a code and the code remains at the study centre. The study centres may require a data transfer agreement with UniversitätsSpital Zürich (USZ).

### Data availability

All data needed to evaluate the conclusions in the article are present in the article. The code of the HFO detector is freely available at the repository https://github.com/ZurichNCH/Automatic-High-Frequency-Oscillation-Detector. Further resources are indexed at https://HFOzuri.ch/

## Analysis plan

### Data management

The researchers from the study sites transform their iEEG datasets to the Brain Imaging Data Structure format which includes electrode positions.^33–36^ The patient code has the format xxxyyy, where xxx is the study centre (Table 1, e.g. 007) and yyy is the centre’s patient number (e.g. 028). Study centres transfer their data sets via https://transfer.usz.ch/to the server maintained by USZ where the analysis is performed. The researchers at USZ document patients and analysis results in a dedicated FileMaker® database. All data remain the property of the contributing centre.

### High-frequency oscillation analysis is blinded to seizure outcome

Researchers at USZ perform HFO analysis. The results of the HFO analysis are communicated to the study centres, where they are set in relation to the RV and the seizure outcome. Special care will be taken that the researchers at USZ are not informed about the seizure outcome of the patients, where iEEG was recorded at Schweizerisches Epilepsie-Zentrum.

### Electrode types and implantation sites

Subdural grid electrodes as well as depth electrodes have been placed according to the findings of the non-invasive presurgical evaluation. Pre-implantation MR and post-implantation MR images or CT images are used to locate each electrode contact anatomically using an intracranial electrode localization and visualization toolbox at the study centre. Study centres account for the the co-registration bias and brain sagging. As a possible method, the electrode positions with respect to the rim of the RV can be determined from post-resection MR coregistered to pre-implantation MR scans.^35,37,38^ This co-registration method accounts for brain deformities both for depth electrodes and grid electrodes. Electrode contacts in white matter are not considered separately in the algorithm.For grid electrodes, the study centres can also compare photographs of the electrode placement with photographs of the RV.

### Data preprocessing

We select data that are recorded during nights while the patients are in NREM sleep. The study centres identify the periods of NREM sleep by polysomnography or iEEG delta power where polysomnography is not available. This reflects current clinical practice in iEEG analysis. Each centre provides the sampling rate and anti-aliasing filter settings during recording of their data. If needed, the data is down-sampled to 2000 Hz using the polyphaser anti-aliasing filter in Matlab. The iEEG is transformed to bipolar channels. The researchers at USZ divide the data into 5 min epochs and inspect the data for persistent artefacts. Channels with persistent artefacts are rejected in the respective 5 min epoch. All 5 min epochs from all nights enter subsequent analysis.

### Definition of a clinical relevant high-frequency oscillation

The HFO detector incorporates information from both time and frequency domain and operates in two stages. In the first stage—baseline detection—the Stockwell transform identifies high entropy segments with low oscillatory activity.^39^ The values of the envelope of the signal at these high entropy segments define the baseline. The second stage – HFO detection – is conducted separately for ripples (band-pass 80–240 Hz, stopband 70 Hz and 250 Hz, FIR equiripple filter with stopband attenuation 60 dB) and fast ripples (FRs) (band-pass 250–490 Hz, stopband 240 Hz and 500 Hz, Table 2). Events with the envelope of the filtered signal exceeding the amplitude threshold for at least 20 ms (10 ms) are labelled as ripples (FR). The threshold is defined as a percentile of the cumulative distribution of the amplitude of the Hilbert envelope taken for baseline segments.^21^ This threshold is the maximum amplitude accepted for an HFO, and it is the same for ripple and FR detection (ThrHilbEnv). HFO not having a minimum of 6 consecutive peaks greater than a threshold are rejected. The threshold is chosen at a percentile of the cumulative distribution of the band-passed signal for baseline segments (ThrFiltRipple and ThrFiltFR, different for ripple and FR detection). The filter thresholds (ThrFiltRipple and ThrFiltFR) are an upper limit for the amplitude for consecutive peak detection. The algorithm then identifies FRs overlapping with a ripple, which we define as a third type of HFO: FR co-occurring with ripples (FRandR). It is certainly possible that ripple and FR can coexist as harmonics/subharmonics of each other but our initial approach to find this biomarker was data-driven (Table 2 in Ref.^17^). The automated HFO algorithm has been proven to efficiently detect clinically relevant HFO and to discard transient artefacts.^17,21,22^ There is no manual rejection of events in this fully automated algorithm.

**Table 2:** Parameters of the detector

	Frequency band	Amplitude threshold	Duration threshold	Filter threshold
**Ripples**	80–250 Hz	ThrHilbEnv = 500	20 ms	ThrFiltRipple = 30
**Fast Ripples**	250–500 Hz	ThrHilbEnv = 500	10 ms	ThrFiltFR = 20

### Definition of the high-frequency oscillation area by rate thresholding

**Figure 1:**
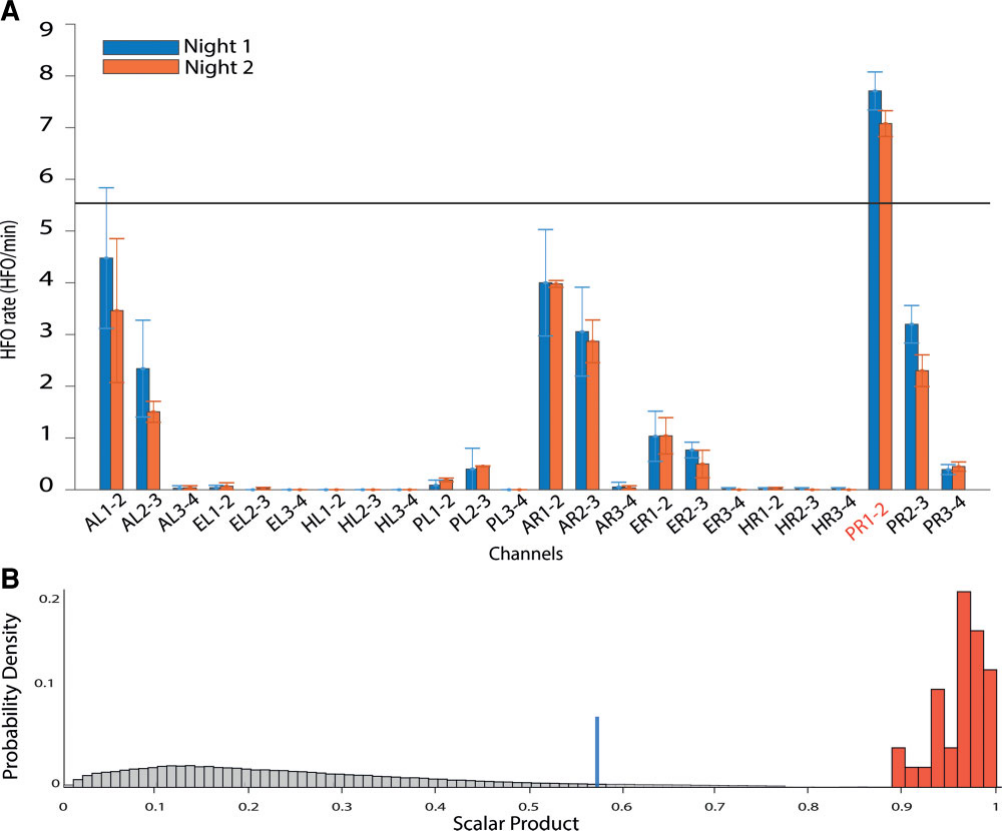
**HFO rate distribution and scalar product.** (**A**) HFO rate (FRandR, co-occurring ripple and fast ripple, HFO/min) from two nights. Standard error bars indicate variability across intervals within nights. Channels with rates that exceed the 95th percentile (HFO rate = 5.6 HFO/min) are candidates to be included in the HFO area (rate thresholding). (**B**) The anatomical distribution of HFO is not random. The true distribution of the normalized scalar product of HFO rates for each pair of intervals (scalar product > 0.8). The random distribution of the normalized scalar product of HFO rates obtained by permutation analysis (scalar product < 0.8, 10000 permutations). The 97.5th percentile of the random permutation (scalar product = 0.57) serves as the significance threshold. 100% of the true distribution exceed the significance threshold; therefore, the anatomical distribution of HFO is not random. PR, posterior hippocampus right.

In the following, we investigate only the FRandR and refer to them as HFO for simplicity. In each recording epoch, we compute the HFO rate by dividing the HFO count per channel by the duration of the epoch in minutes. We then analyze the spatial distribution of HFO rates across channels in each patient. The ensemble of the channels whose rates exceed the rate threshold (95th percentile of the HFO rate distribution) is defined as the HFO area (Fig. 1A).

### Reliability of the spatial distribution of the high-frequency oscillation area

We then test whether the HFO area is simply a product of chance. For each epoch, we construct a HFO vector where each recording channel represents a dimension and the HFO rate on that channel represents the length in that dimension. We select all pairs of HFO vectors within the night from different epochs and compute the normalized scalar product of the spatial distribution of the HFO rates. The scalar product is 1 for perfectly overlapping spatial distributions of HFO rate and lower otherwise. To test the magnitude of the true scalar product against chance, we construct a distribution of scalar products by randomly permuting (*N* = 10 000) the order of channels for each epoch (Fig. 1B). The true value of the scalar product is considered statistically significant if it exceeds the 97.5% percentile of the distribution. We construct the scalar product distribution based on HFO vectors from all channel-epoch pairs and we test it against chance only once. In this way, we do not perform multiple comparisons. We are aware that with our approach using relative thresholds (percentiles) we will always delineate a HFO area, which has to be validated further.

### Temporal consistency of the spatial distribution of the high-frequency oscillation area

**Figure 2:**
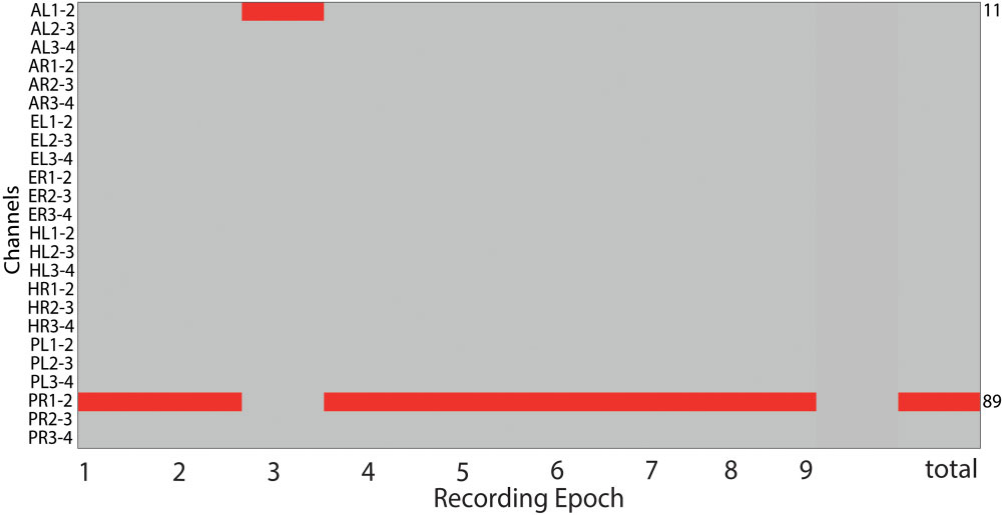
**Temporal consistency of HFO rates.** Reproducibility of the HFO area over 5 min epochs. Horizontal bars denote channels where the HFO rate exceeds the 95th percentile in that interval. The channel from the tip of recording electrode PR has red bars for a dwell time = 89% of the recording epochs. The second but last column guides the eye. The last column illustrates the total of the channels that meet the 95% criterion. PR, posterior hippocampus right.

**Figure 3:**
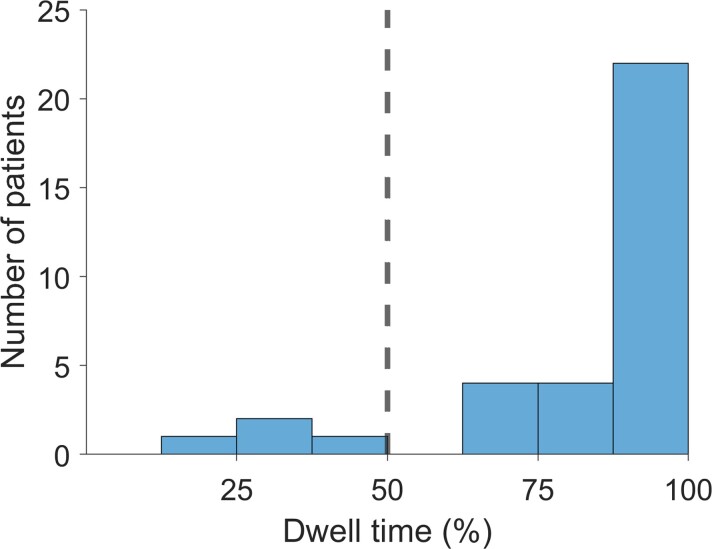
**Dwell time distribution.** The histogram of dwell times obtained from the pooled cohort (*N* = 34) of the previous studies.^17,22^ Patients with HFO area with dwell time <50% were clearly separated from the patients with HFO area ≥50% dwell time.

As a final and crucial step in our algorithm, we quantify the temporal consistency of the HFO area over the ensemble of recording epochs by counting the percentage of epochs that each channel spends in the HFO area (dwell time) for a given patient (Fig. 2). The channels with <50% dwell time are not considered further. The channels with ≥50% dwell time constitute the HFO area. In the pooled data of our previous studies,^17,22^ the median (IQR) percentage of channels in the HFO area was 3.9% (3.5%, 4.8%). We assume that the prediction of the seizure outcome is only valid if the HFO area is stable over time. If the dwell time remains <50% for all channels, we consider the HFO area an unstable measure and we cannot make a recommendation on that particular patient.^17,22^ Conversely, we will only recommend removing the HFO area in the patient if the temporal consistency over the patient’s 5 min epochs reaches ≥50%. In two previous studies,^17,22^ in the distribution of patients, patients with HFO area with dwell times <50% were clearly separated from those patients with ≥50% dwell time (Fig. 3). We are aware that this constraint is excluding patients who might have multiple foci and we include only those patients where we are looking to resect a single focus. As an important result of the study, we will report the proportion of patients to whom the method is applicable (dwell time ≥ 50%). We will also report this proportion after subdividing the patient group according to the type of electrodes implanted. In the pooled data from our two previous studies^17,22^ the proportion of patients that did not meet the dwell time criterion was 5/16 for grid patients and 0/22 for depth patients (*P* = 0.0049, χ^2^ test).

### Using the high-frequency oscillation area to predict seizure outcome

In the study design, the primary outcome is seizure freedom (ILAE 1). Automated HFO detection and analysis are blind to clinical information. Electrodes landing on the rim of the resection are deemed to be part of the RV. We define as true positive (TP) a patient where the HFO area is not fully located within the RV, i.e. at least one channel of the HFO area is not resected and the patient suffers from recurrent seizures (ILAE 2–6). We define as false positive (FP), a patient where the HFO area is not fully located inside the RV but who achieves seizure freedom (ILAE 1). We define as false negative (FN), a patient where the HFO area is fully located within the RV but who suffers from recurrent seizures. We define as true negative (TN), a patient where the HFO area is fully located inside the RV and who becomes seizure-free. The positive-predictive value is calculated as PPV = TP/(TP + FP), negative-predictive value as NPV = TN/(TN + FN), sensitivity = TP/(TP + FN), specificity = TN/(TN + FP) and accuracy = (TP + TN)/N. We use these values as the elements of the confusion matrix.

### High-frequency oscillation in the normal human brain

Since physiological HFO have been detected in the normal human brain, in particular in sensory-motor and occipital areas,^26^ we investigate whether the target of this study (FRandR) occurs more frequently in sensory-motor or occipital areas. Patients will be assigned TP, TN, FP or FN regardless of whether the HFO area occurs in sensory-motor or frontal or occipital areas, i.e. this will not influence the validation of the algorithm. In a *post hoc* analysis, we will document whether FRandR occur in sensory-motor or frontal or occipital areas more often than expected by chance and whether this actually impairs the biomarker ability of FRandR.

### Study schedule

The study starts on 1 March 2022. The study centres transfer iEEG data within 3 months after the start of the study. HFO analysis is finished on 1 September 2022. The study centres analyse RV and seizure outcome. They provide the list of channels within the RV and the seizure outcome to the researchers at USZ until 1 November 2022. The manuscript is submitted by 1 March 2023.

## Sample size estimation

In the pilot studies^17,22^ we obtained estimates for the derivations of the confusion matrix. In the study on Zurich data (*N* = 20), three patients had median dwell time <50% and would be excluded from further analysis.^17^ In the study on Geneva data (*N* = 16), two patients had median dwell time <50% and would be excluded from further analysis.^22^ Note that these five patients (5/36 = 14%) were all patients with extratemporal lobe epilepsy (ETE) with grid electrodes. The lower limit for the number of patients that must be included before publication is *N* = 255 (Table 1). Table 3 shows the expected 95% CI for the size of the expected multicentre cohort size for *N* = 255 and *N* = 300 patients; all confidence intervals are above chance level (50%).

**Table 3:** Sample size estimation

	Estimate for *N* = 20 + 16	95% CI expected for *N* = 255	95% CI expected for *N* = 300
Specificity	88%	(83% 92%)	(84% 91%)
Sensitivity	76%	(70% 81%)	(71% 81%)
NPV	79%	(73% 84%)	(74% 83%)
PPV	87%	(82% 91%)	(83% 91%)
Accuracy	82%	(77% 86%)	(77% 86%)

The derivations from the confusion matrix are shown as a scenario for cohort size *N* = 255 (the minimum patient number from Table 1) and *N* = 300. We use as basis the values obtained after combining the two pilot cohorts with *N* = 20 (Ref.^17^) and *N* = 16 (Ref.^22^) to estimate the confidence intervals.

## Abbreviations

CI =: confidence interval
ETE =: extratemporal lobe epilepsy
FN =: false negative
FP =: false positive
FR =: fast ripple
FRandR =: fast ripple co-occurring with ripple
HFO =: high-frequency oscillation
IED =: interictal epileptic discharge
iEEG =: intracranial EEG
ILAE =: International league against epilepsy classification
NPV =: negative-predictive value
NREM =: non-rapid eye movement
PPV =: positive-predictive value
RV =: resection volume
SNR =: signal-to-noise ratio
SOZ =: seizure onset zone
TN =: true negative case
TP =: true positive case
USZ =: UniversitätsSpital Zürich
